# Editorial: Metastatic Castration Resistant Prostate Cancer: Prognosis and Treatment

**DOI:** 10.3389/fonc.2022.913630

**Published:** 2022-05-13

**Authors:** Sakthivel Muniyan, Benyi Li, Surinder K. Batra

**Affiliations:** ^1^ Department of Biochemistry and Molecular Biology, University of Nebraska Medical Center, Omaha, NE, United States; ^2^ Department of Urology, University of Kansas Medical Center, Kansas City, KS, United States; ^3^ Fred and Pamela Buffett Cancer Center, University of Nebraska Medical Center, Omaha, NE, United States; ^4^ Eppley Institute for Research in Cancer and Allied Diseases, University of Nebraska Medical Center, Omaha, NE, United States

**Keywords:** mCRPC, androgen receptor, ARv7, molecular diagnosis, 77Lu-PSMA-I&T, PSA, targeted therapy

Prostate cancer (PCa) continues to be one of the leading causes of cancer-related deaths in men worldwide (1). Localized PCa responds well with the initial treatment such as surgery and/or radiation therapy; however, the tumor recurred in ~50% of the patient population (2). Biochemically recurrent or advanced PCa is clinically managed with androgen deprivation therapy (ADT) (3). However, advanced PCa progresses into metastatic castration-resistant phenotype in almost all patients. As a standard clinical practice, patients with metastatic castration-resistant prostate cancer (mCRPC) have been treated with chemotherapies such as mitoxantrone and docetaxel until 2010 (3). Technological advancements in the past two decades led to a greater understanding and revealed that residual androgens, ADT-induced androgen receptor (AR) splice variants, AR mutations, and growth factor signaling-mediated AR activation are common underlying mechanisms of mCRPC. In parallel, eleven different therapeutic agents have been approved for mCRPC, and many are AR signaling inhibitors. Also, technological advancement led to new biomarkerrs and novel targeted therapies in the management of advanced PCa (**Figure 1**).

**Figure 1:**
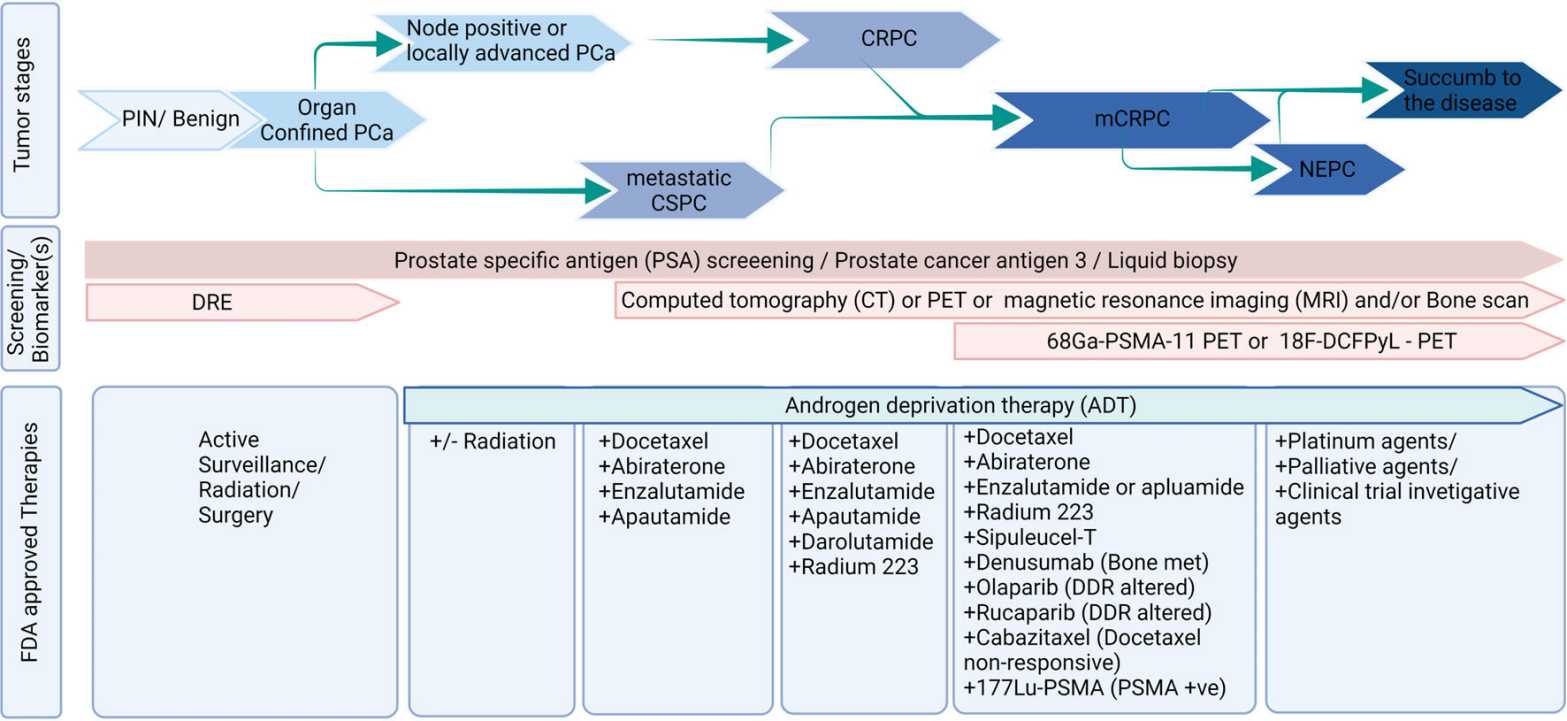
Diagnosis and treatment landscape of prostate cancer. Figure was created using BioRender.com.

Treating PCa patients with subsequent androgen receptor (AR) signaling targeting agents, next-generation chemotherapeutic agents, bone-seeking agents, and PARP inhibitors extends the survival for a few months (Garje et al.; 3). Still, CRPC patients have poor outcomes, with most patients dying within two years of diagnosis (4). These results left many lacunae and suggestions, (i) identification of the optimal sequence of available agents or preference of treatment over others in a subset of the population; (ii) a predictive biomarker that can inform which subset of patients can benefit from therapy; (iii) identification of a novel treatment that can increase the survival of mCRPC patients; and (iv) a way to improve the quality of life (QoL) among PCa survivors. This special issue focuses on presenting original articles and systemic reviews from experts and clinical investigators in PCa that cover advanced treatment options, biomarkers, and quality of life (QoL) in the landscape of mCRPC (**Figure 1**).

Molecular diagnosis of tumor samples has become increasingly recognized and important with the development of various therapies for mCRPC. Furthermore, one of the best approaches to treating mCRPC is to predict the patient subgroup who would be eligible for a particular therapy. Although PSA screening has had a paradigm-changing impact on PCa identification, treatment, and management, additional biomarkers are necessary in mCRPC due to tumor heterogeneity and AR-negative tumors in a subset of patients. One such emerging non-invasive biomarker is ARv7, which can be detected in tumor samples, serum, and circulating tumor cells. The meta-analysis by Wang et al. assessed the prognostic value of ARv7 in mCRPC patients. This meta-analysis evaluated 21 clinical studies for the prognostic value of AR-V7 in patients with mCRPC treated with AR pathway inhibitors or taxane chemotherapies. The meta-analysis revealed significant differences between AR-V7 positive and AR-V7 negative patients for mCRPC treatment, with AR-V7 positive patients receiving AR signaling inhibitors having a shorter PSA response rate, shorter PFS, and shorter OS than those receiving chemotherapy. In comparison, the difference is nonsignificant in AR-V7-negative patients. The technological advances reached a new high in every aspect, and the assay’s sensitivity allowed to profile the molecular changes using a minimal quantity of samples. Considering the aberrant AR signaling, Hamid et al. determined the AR signaling alterations using multiple approaches in CRPC tumor samples. The authors performed IHC analysis to determine AR protein levels in patient samples. Gene expression was measured using the SYBR-based qRT-PCR method. AR mutational analyses were performed with exon-specific primers. This study revealed that most patients had intense levels of AR protein with N-terminal antibodies and reduced expression with C-terminal antibodies. The AKR1C3 gene, which helps in intratumor steroidogenesis, was explicitly expressed in a subgroup of patients. AR amplification was observed only in the AR high group. The AR-variants are relatively higher in the nuclear AR high subgroups. This study reaffirms that AR signaling is still active in CRPC, and molecular characterization of tumor samples could help stratify and treat patients (Hamid et al.)

Irrespective of the treatment modalities, most CRPC patients develop bone metastasis. The metanalysis by Tong et al.  assessed the prognostic value of skeletal-related parameters in mCRPC overall survival. This study examined the association between alkaline phosphatase, bone-specific alkaline phosphatase, urinary N-telopeptide, bone scan index, and overall survival in patients with metastatic PCa. The analysis revealed that higher levels of ALP, BSAP, and uNTx, progression of BSI, as well as BPI-SF scores were associated with lower OS in randomized controlled trials published between 2010 and 2019. Considering the ambiguity of the PSA value in mCRPC, bone-related parameters, AR variants, and, more importantly, CTC can help stratify the risk of mCRPC patients before the start of treatment.

As two-thirds of PCa patients die of other complications (5), delaying the progression of PCa in some way would decrease prostate cancer-specific mortality (PCSM). By testing the hypothesis that the delayed progression of metastatic hormone-sensitive prostate cancer (mHSPC) to CRPC may increase overall survival (OS), Wenzel et al. retrospectively evaluated the association between time to castration resistance (TTCR) in mHSPC patients and overall survival (OS). Among the 204 eligible patients from University Hospital Frankfurt, TTCR of less than 12 months was associated with the worst OS in mHSPC patients. The patients who took more than 24 months to develop castration resistance had the most prolonged OS. Based on the observation, the study suggests that TTCR could affect OS in patients with mHSPC (Wenzel et al.).

With the growing treatment arsenal, currently, there are limited directions for the optimal sequencing of available therapies for mCRPC. In the article by Briones et al. retrospectively analyzed the effectiveness of docetaxel (DOC) and abiraterone (ABI) in patients with metastatic castration sensitive prostate cancer (mCSPC) progression-free survival (PFS) in a real-world scenario. The authors analyzed 121 mCSPC patients treated in a single institution between December 2014 and March 2021. Seventy-nine patients were treated with DOC, and forty-two patients were treated with ABI. The primary endpoint was PFS, and the secondary endpoint was OS. The median survival for men with mCSPC was 39.6 months for the DOC cohort and 25.1 months for the ABI cohort. The ABI group had significantly longer median survival than the DOC cohort (Briones et al.).

Finally, in this Research Topic, Bu et al. studied the response of the investigational agent (now approved by FDA) 77Lu-PSMA-I&T radioligand in patients with mCRPC from East Asia. In this prospective single-arm study, the authors followed the treatment response and treatment-related toxicity in 40 patients. Among patients with followable criteria, 25 patients had a partial response (PSA decline ≥50%) and no response (stable disease) in five patients, and the rest (10 patients) had progressive disease. Additionally, a subset of patients underwent a ^68^Ga-PSMA-11 PET/CT scan to determine their response to treatment. However, the authors did not observe any association between ^68^Ga-PSMA-11 imaging and response, possibly due to a limited cohort of patients, and not all patients underwent a ^68^Ga-PSMA-11 PET/CT scan. Overall, the authors observed that 177Lu-PSMA-I&T had a better response and was tolerable in patients with mCRPC.

Since 2010, the new treatment agents for advanced PCa have had a paradigm-shifting impact on the survival of CRPC patients. In addition to the article covered in this special issue, recently, PARP inhibitors are gaining momentum in the space of mCRPC patients with DNA damage repair pathway alterations. Emerging evidence shows that an upfront combination of ADT with other established treatments such as radiation, chemotherapy, and second-generation AR pathway inhibitors had better PFS and OS among CRPC patients. Further, novel combinations might delay the progression of PCa and the treatment agents with known pharmacokinetics would lead to better therapeutic effects with lesser treatment-related toxicity (6). As these novel treatment approaches continue to improve the survival outcomes among PCa patients, newer treatments such as ^177^Lu-PSMA-617, immunotherapy, and protein degraders are expected to show increased survival outcomes in mCRPC.

We thank the authors for the excellent research and review articles contribution to this special issue. We hope that the information that appeared in this issue will be informative and helpful to the scientific community.

## Author Contributions

SM, BL, and SB contributed to writing the manuscript. All authors contributed to the article and approved the submitted version.

## Funding

The authors of this report preparation were supported, in parts, by the Department of Defense Grants (W81XWH-18-1-0308 to SB and W81XWH2010637 to BL) and the National Institutes of Health (NIH) Grant (U01 CA185148 to SB).

## Conflict of Interest

SB is one of the founders of Sanguine Diagnostics and Therapeutics, Inc.

The remaining authors declare that the research was conducted in the absence of any commercial or financial relationships that could be construed as a potential conflict of interest.

## Publisher’s Note

All claims expressed in this article are solely those of the authors and do not necessarily represent those of their affiliated organizations, or those of the publisher, the editors and the reviewers. Any product that may be evaluated in this article, or claim that may be made by its manufacturer, is not guaranteed or endorsed by the publisher.
